# Hair Growth-Promoting Activity of a Biorenovated Esculetin Complex Through Modulation of Dermal Papilla Cell Function and Hair Follicle Cycling

**DOI:** 10.3390/ph19091430

**Published:** 2026-09-10

**Authors:** Yu Jin Lee, Yeo Jeong Han, Je Joung Oh, Hyehyun Hong, Hyun Min Ko, Seung-Young Kim, Ji Hoon Jung

**Affiliations:** 1 College of Korean Medicine, Kyung Hee University, Seoul 02447, Republic of Korea; yujin3347@naver.com (Y.J.L.); spring10412@naver.com (Y.J.H.); jezzzje9@gmail.com (J.J.O.); 2 Department of Pharmaceutical Engineering & Biotechnology, Sunmoon University, Asan 31460, Republic of Korea; kongjjo123@naver.com; 3 Department of Genetics, LSU Health New Orleans School of Medicine, Louisiana State University, New Orleans, LA 70112, USA; rhgusals93@naver.com

**Keywords:** hair growth, biorenovated esculetin complex (BEC), hair follicle

## Abstract

**Objectives**: Hair loss is a prevalent condition influenced by genetic, hormonal, and environmental factors, and effective therapies with minimal side effects are highly desired. In this study, the hair growth-promoting potential of a biorenovated esculetin complex (BEC) was evaluated in a C57BL/6N mouse model. **Methods**: BEC was topically applied to the depilated dorsal skin of mice once daily for 24 days. Hair growth was evaluated by measuring hair length, thickness, and weight, together with histological assessment of hair follicle number, follicle depth, and dermal thickness. Protein expression associated with Wnt/β-catenin and AKT signaling was evaluated by Western blot analysis. **Results**: BEC treatment accelerated hair regrowth and increased hair length, thickness, and weight compared with the vehicle control under the present experimental conditions. Histological analysis of hematoxylin-and-eosin-stained skin sections showed increased hair follicle number and greater follicle depth in the BEC-treated group, whereas dermal thickness was not significantly altered. Western blot analysis showed changes in β-catenin, phospho-GSK-3β (Ser9), and PCNA levels following BEC treatment. The p-AKT/AKT ratio also showed an increasing trend, although the difference was not statistically significant. **Conclusions**: These findings demonstrate the hair growth-promoting activity of the BEC preparation in this short-term murine model and suggest that these effects are associated with changes in Wnt/β-catenin- and AKT-related signaling. Further studies are warranted to characterize the active components, dose dependence, mechanism of action, and safety profile of BEC.

## 1. Introduction

Hair is a critical skin appendage that performs essential physiological functions, including protecting the scalp from external physical and chemical stimuli and contributing to thermoregulation. Beyond its biological utility, hair plays a pivotal role in personal appearance and identity [1,2,3]. The functionality of hair is governed by the formation of hair follicles, which maintain hair growth through a tightly regulated cycle of anagen (growth), catagen (regression), and telogen (resting) phases [4,5]. This cycle is modulated by complex interactions between follicular cells, growth factors, and the surrounding microenvironment. When this balance is disrupted—often resulting in a shortened anagen phase and a prolonged telogen phase—hair’s regenerative capacity declines, leading to the pathological condition known as alopecia [6,7,8].

Recent research emphasizes that alopecia is not merely a consequence of aging but can reflect disruption of the follicular microenvironment by genetic and environmental factors, including oxidative stress and chronic inflammation [9,10,11,12,13]. Although FDA-approved treatments such as minoxidil and finasteride are widely used, their long-term use may be limited by adverse effects or variable treatment responses [14,15,16].

Consequently, there is continued interest in naturally derived compounds with hair growth-promoting activity and favorable tolerability.

Esculetin (6,7-dihydroxycoumarin) is a natural phenolic compound known for its potent anti-inflammatory and anti-aging properties. However, its therapeutic application has been hindered by low solubility, potential cytotoxicity at high concentrations, and inconsistent efficacy in transitioning from in vitro to in vivo models [17,18,19,20]. To overcome these limitations, biorenovation technology—a bioconversion approach using microbial catalysts—was employed to generate a biorenovated esculetin complex (BEC). Previous studies have demonstrated that biorenovation can modify the physicochemical and biological properties of natural compounds [21,22]. However, the effects of BEC on hair follicle dynamics remain unclear.

The present study therefore aimed to evaluate the hair growth-promoting activity of BEC in a C57BL/6 mouse model. Hair regrowth, hair morphometric parameters, and histological changes in hair follicle number and depth were assessed following topical BEC treatment. In addition, changes in Wnt/β-catenin- and AKT-related signaling proteins were examined to identify molecular changes associated with the observed hair growth response. These findings provide experimental evidence for the hair growth-promoting activity of BEC and support further investigation of its biological effects on hair follicle growth.

## 2. Results

### 2.1. Effect of BEC on Hair Growth in C57BL/6 Mice

**Figure 1:**
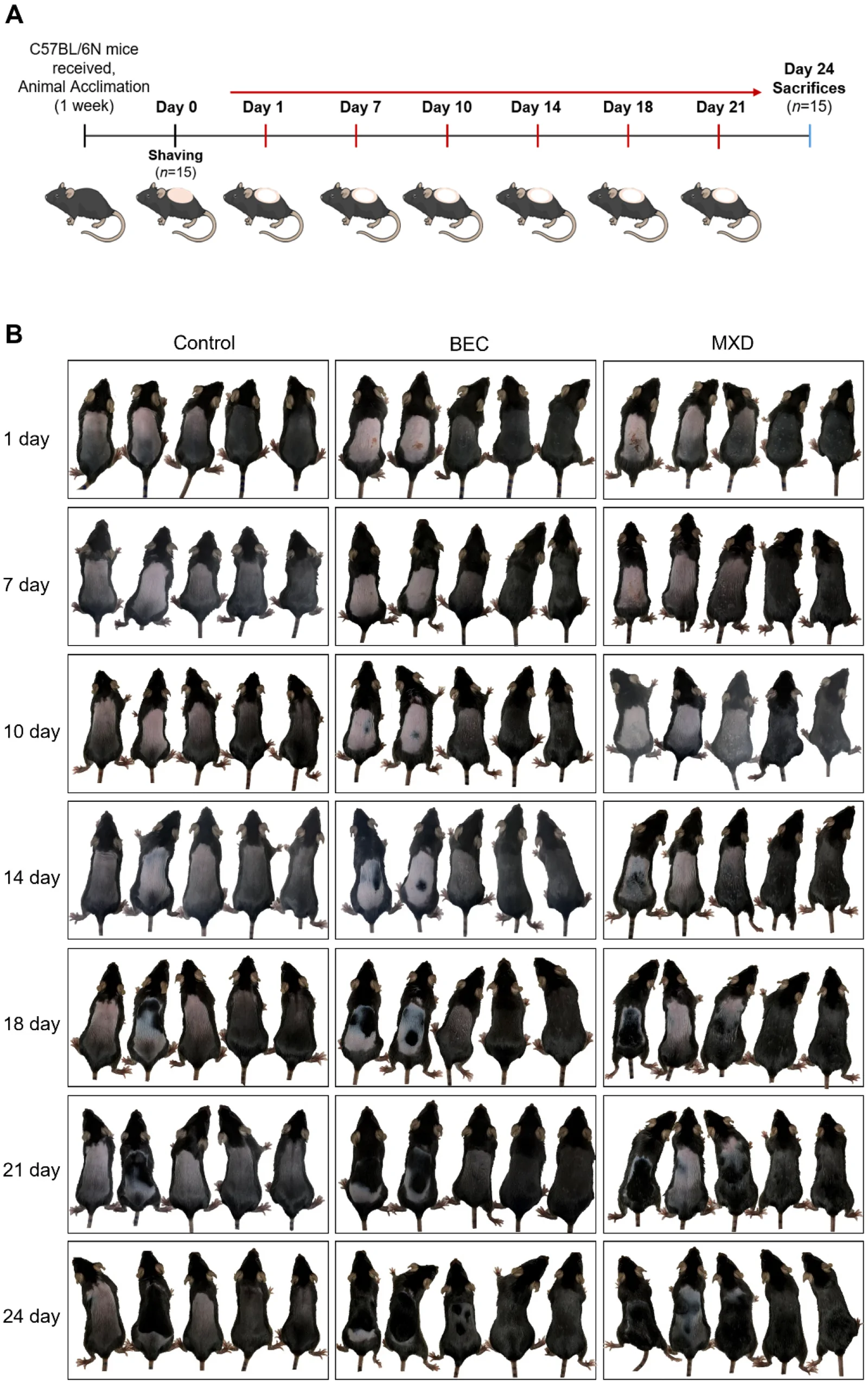
Effect of BEC on hair growth in C57BL/6 mice. (**A**) After a 7-day acclimatization period, six-week-old male C57BL/6 mice were randomly assigned to groups (*n* = 5 per group): vehicle control, BEC (20 μg/mL), or Minoxidil (20 μg/mL). Each group received a daily topical application of 40 mg of the assigned treatment to the depilated dorsal skin. (**B**) Digital images were recorded during the treatment period to monitor hair regrowth.

To evaluate the hair growth-promoting effect of BEC in vivo, depilated dorsal skin of six-week-old male C57BL/6 mice was topically treated once daily for 24 days with vehicle (control), BEC (20 μg/mL), or minoxidil (MXD, 20 μg/mL). Hair growth progression was visually monitored and recorded using digital imaging throughout the experimental period. No noticeable differences in hair regrowth were observed among the groups up to day 7. From day 14, gradual hair regrowth was observed in both the BEC- and MXD-treated groups (Figure 1A,B). By day 18, limited localized hair regrowth was observed in some animals in the vehicle control group; however, the extent and density of regrowth were lower than those observed in the BEC- and MXD-treated groups. At the end of the experiment (day 24), both BEC- and MXD-treated groups exhibited extensive hair regrowth across the dorsal area. Although inter-individual variability in regrowth was observed, the overall pattern supported further quantitative analysis of hair weight, length, thickness, and follicular structure.

### 2.2. Effect of BEC on Hair Weight and Length in a Mouse Model

**Figure 2:**
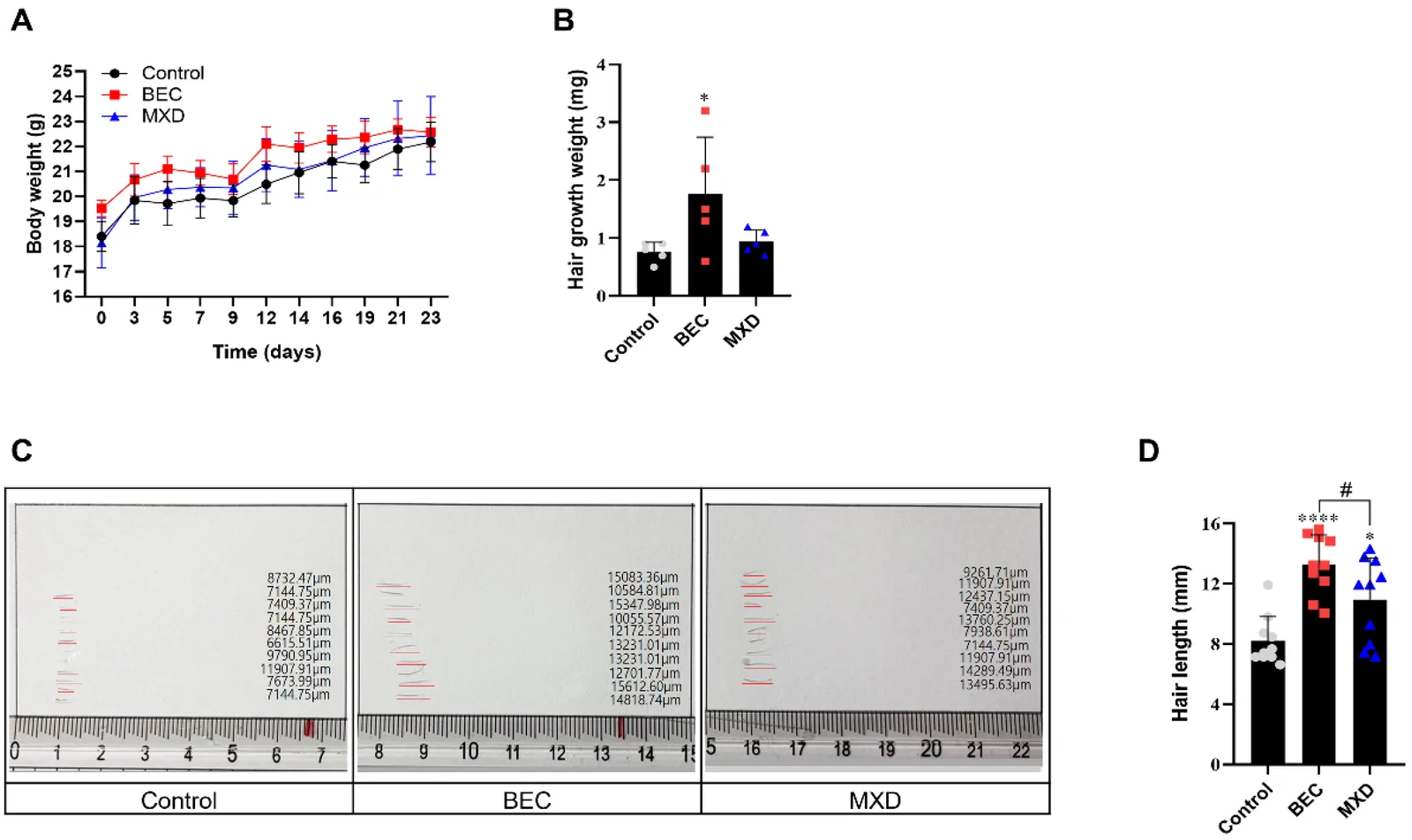
Effect of BEC on body weight, hair weight and hair length. (**A**) Body weight was measured every two days throughout the experimental period. (**B**) On day 24, regrown hair was collected from the dorsal skin and weighed using an analytical balance (PX224KR/E, OHAUS Corporation, Parsippany, NJ, USA). (**C**) Representative microscopic images of hair samples collected on day 24. (**D**) Quantification of hair length. Data are expressed as the mean ± SD (*n* = 5). * *p* < 0.05, **** *p* < 0.0001 vs. the vehicle control group. # *p* < 0.05 vs. the Minoxidil group. BEC, biorenovated esculetin; MXD, minoxidil.

Hair samples were collected on day 24, and hair weight and length were measured to quantitatively assess the hair growth-promoting effect of BEC. No significant changes in body weight were observed among the groups during the experimental period (Figure 2A). Hair weight was significantly increased in the BEC-treated group compared with the control group, although inter-individual variability was observed. A statistically significant difference was also observed between the BEC- and MXD-treated groups under the conditions tested (Figure 2B). Hair length was significantly increased in the BEC-treated group, consistent with visual observations (Figure 2C and Figure S4). These findings indicate that BEC treatment increased hair weight and length in this mouse model.

### 2.3. Effect of BEC on Hair Thickness in a Mouse Model

**Figure 3:**
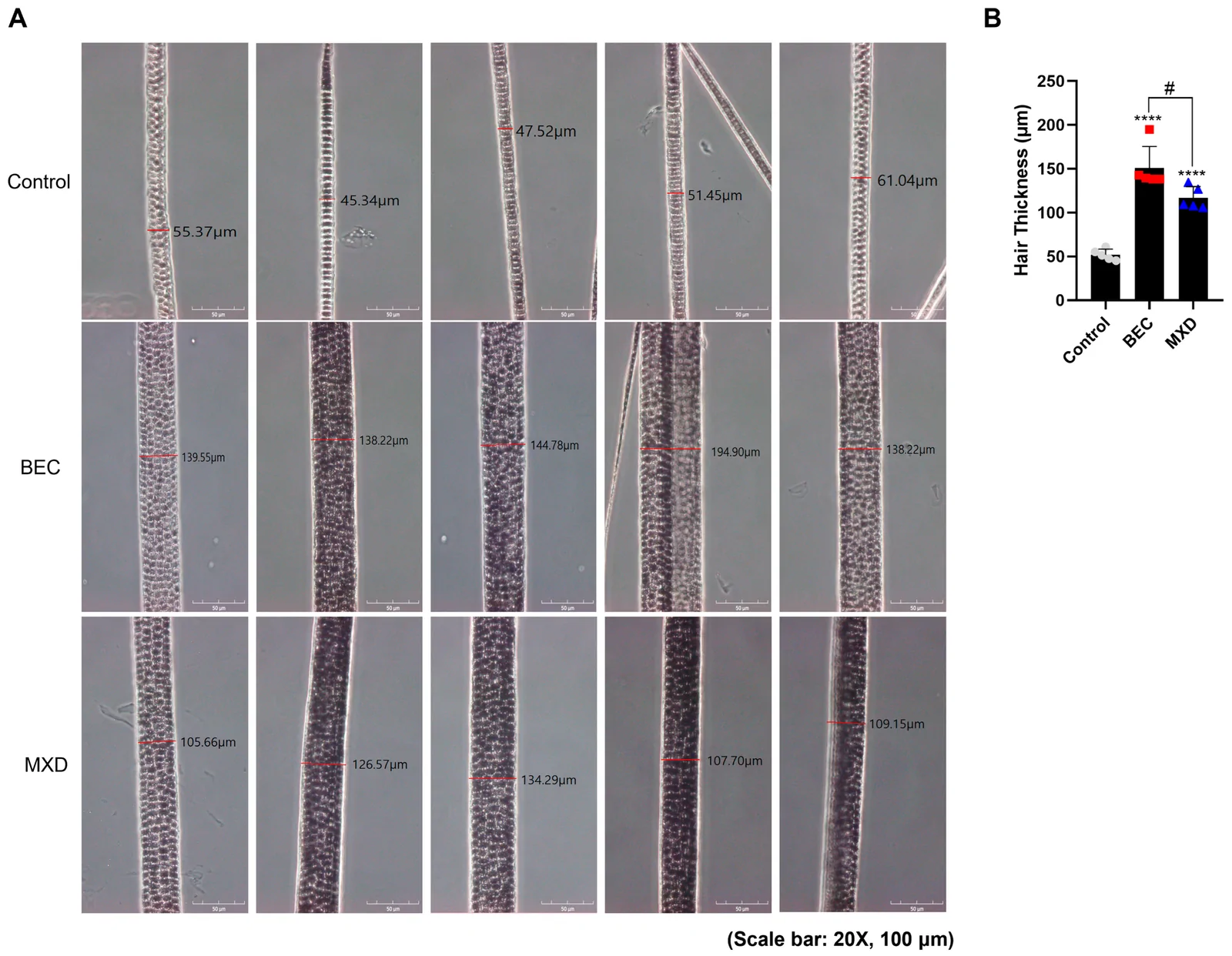
Quantitative and qualitative analysis of hair thickness. (**A**) Representative microscopic images of hair samples collected on day 24. (**B**) Quantification of hair thickness. Under the conditions tested, BEC (20 μg/mL) increased hair thickness relative to the vehicle control and MXD (20 μg/mL) group. Data are expressed as the mean ± SD (*n* = 5). **** *p* < 0.01 vs. the vehicle control group. # *p* < 0.001 vs. the Minoxidil group. BEC, biorenovated esculetin; MXD, minoxidil.

Hair samples were collected on day 24, and hair thickness was analyzed to further evaluate the hair growth-promoting effect of BEC. Although inter-individual variability was observed, hair samples from the BEC-treated group showed a denser structural appearance than those from the vehicle control group (Figure 3A). Quantitative analysis showed increased hair thickness in the BEC-treated group relative to the vehicle control and MXD groups under the present experimental conditions (Figure 3B). These findings indicate that BEC treatment was associated with increased thickness of regenerated hair.

### 2.4. Effect of BEC on Hair Follicle Structure and Dermal Thickness in a Mouse Model

**Figure 4:**
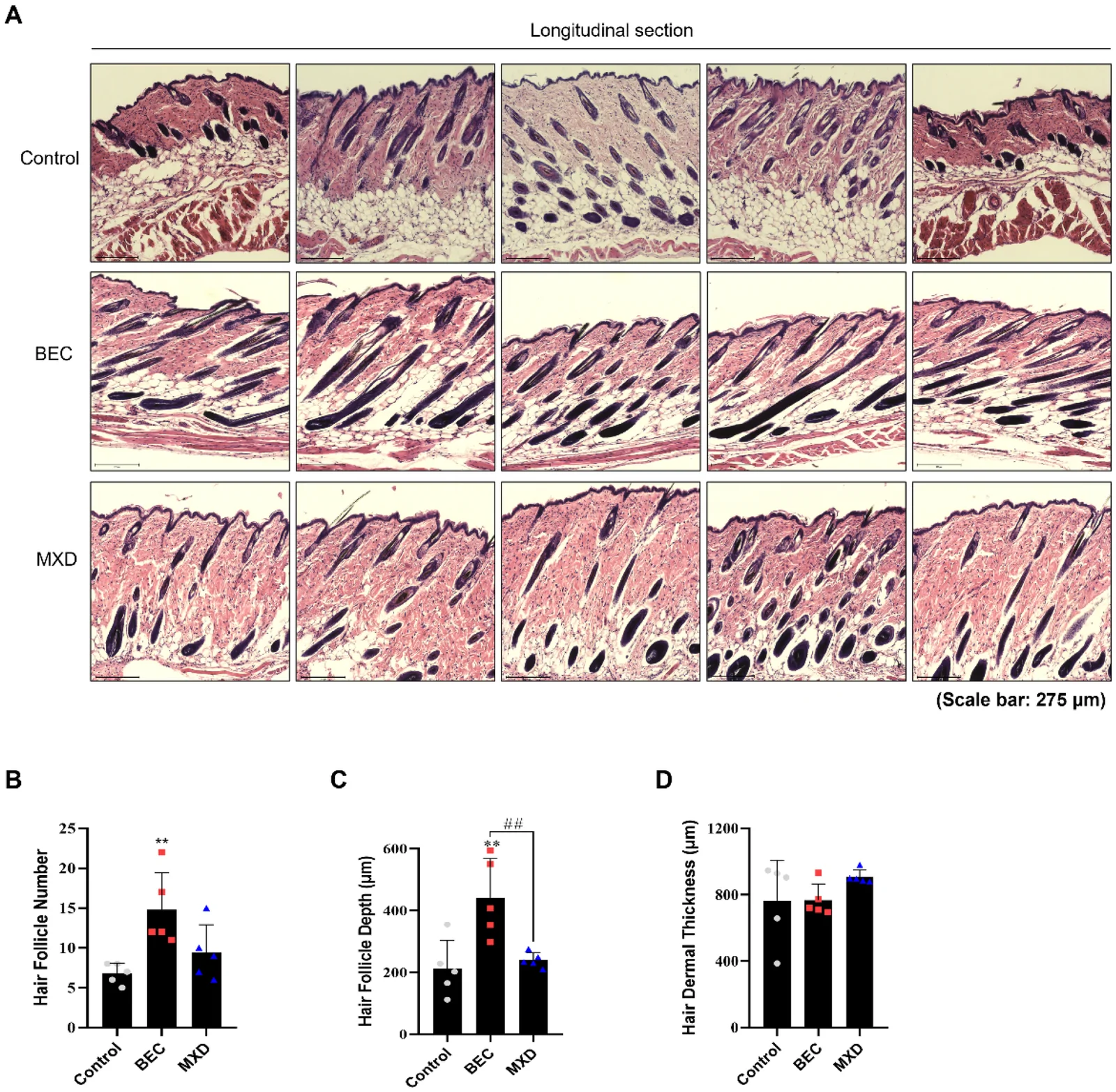
Histological analysis of hair follicle number, follicle depth and dermal thickness. (**A**) Representative images of skin sections stained with hematoxylin and eosin (H&E). (**B**) Quantification of hair follicle number. (**C**) Quantification of hair follicle depth. (**D**) Quantification of dermal thickness. Data are expressed as the mean ± SD (*n* = 5). ## *p* < 0.01 vs. the MXD group; ** *p* < 0.01 vs. the vehicle control group. BEC, biorenovated esculetin; MXD, minoxidil.

To evaluate the effects of BEC on hair follicle development and skin structure in vivo, dorsal skin tissues were collected and subjected to histological analysis following hematoxylin–eosin (H&E) staining. In the BEC-treated group, hair follicles extended deeper into the skin and displayed a more vertically aligned structure than those in the vehicle control group (Figure 4A), morphological features that are consistent with anagen-associated follicular activity. Quantitative analysis showed an increased number of hair follicles in the BEC-treated group compared with the vehicle control group and more follicles in the BEC-treated group than in the MXD-treated group (Figure 4B,C). No significant differences in dermal thickness were observed among the groups (Figure 4D). These results suggest that BEC treatment was associated with changes in hair follicle number and depth without measurable changes in overall dermal thickness.

### 2.5. BEC Treatment Modulates Wnt/β-Catenin and AKT Signaling

**Figure 5:**
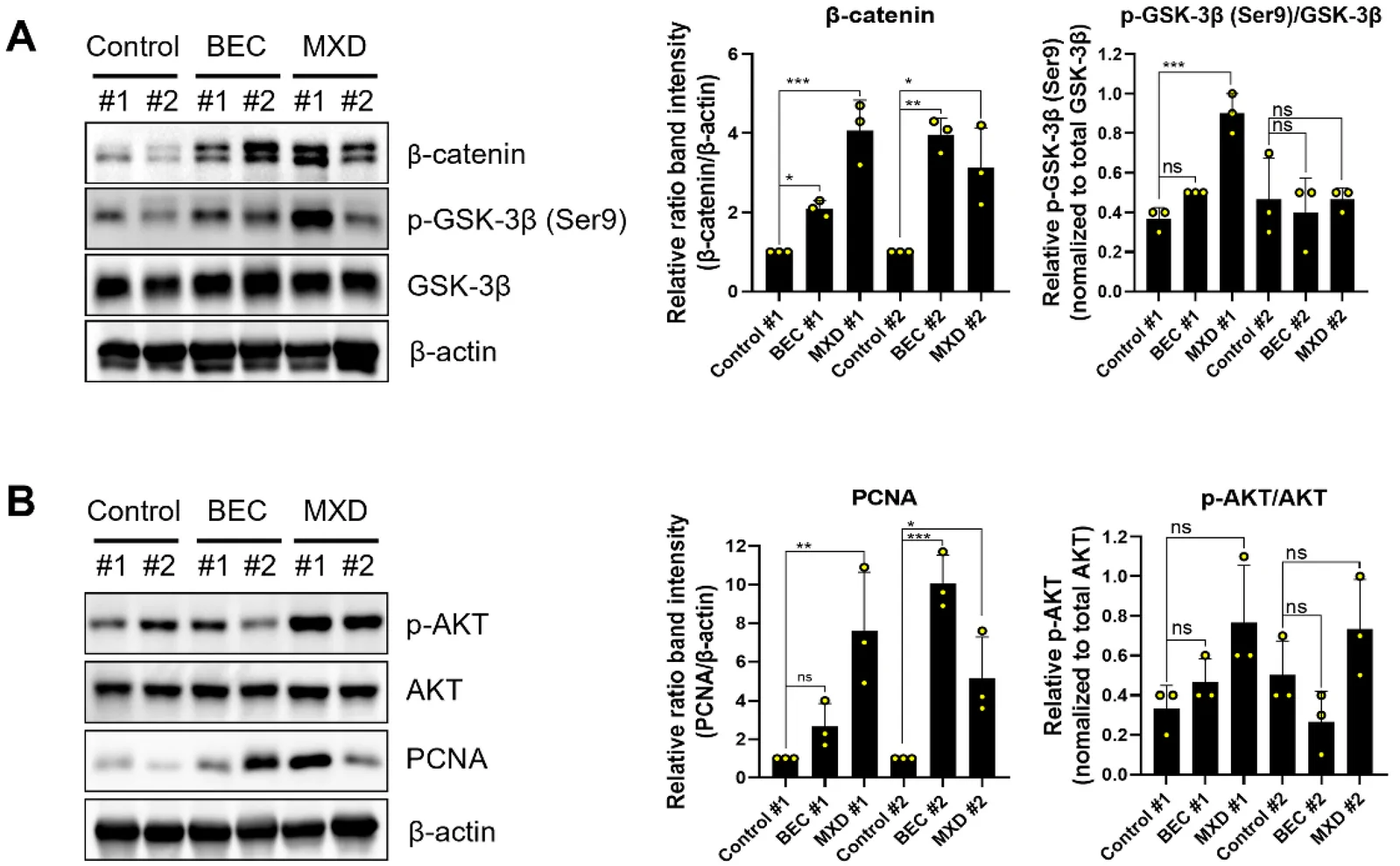
Effects of BEC and MXD on the Wnt/β-catenin- and AKT-related signaling proteins in mouse dorsal skin. (**A**) Western blot analysis of β-catenin, phospho-GSK-3β (Ser9), and total GSK-3β. β-Actin was used as a loading control. Bar graphs show the relative band intensity of β-catenin normalized to β-actin and the ratio of p-GSK-3β to total GSK-3β. (**B**) Western blot analysis of phospho-AKT, total AKT, and PCNA. The bar graphs show PCNA normalized to β-actin and the p-AKT/AKT ratio. Data are expressed as the mean ± SD (*n* = 3). Statistical significance was defined as * *p* < 0.05, ** *p* < 0.01, *** *p* < 0.001 vs. the vehicle control group; ns, not significant. BEC, biorenovated esculetin; MXD, minoxidil.

To examine the molecular mechanisms underlying the hair growth-promoting effects of BEC, Wnt/β-catenin- and AKT-related signaling proteins were analyzed in mouse dorsal skin. Western blot analysis showed that BEC treatment increased β-catenin expression and the level of inhibitory phosphorylation of GSK3β (Ser9) relative to the vehicle control group (Figure 5A). These changes are consistent with modulation of Wnt/β-catenin-related signaling. PCNA, a marker associated with cell proliferation, was also increased in the BEC-treated group (Figure 5B). In addition, the p-AKT/AKT ratio increased following BEC treatment, although the difference was not statistically significant, suggesting that the growth response to BEC was accompanied by alterations in GSK-3β/β-catenin-, AKT-, and PCNA-related markers. Furthermore, inhibitor-based experiments in human follicle dermal papilla cells (HFDPCs) supported the functional involvement of AKT signaling in the cellular response to BEC (Supplementary Figure S5), whereas the contribution of the GSK-3β/β-catenin pathway remains to be functionally established.

## 3. Discussion

In this study, topical BEC treatment promoted hair regrowth in a C57BL/6 mouse model, as demonstrated by increases in hair weight, length, and thickness, together with increases in hair follicle number and depth. BEC treatment did not significantly alter dermal thickness, and the deeper, more vertically oriented follicles observed in the BEC-treated group were consistent with morphological features associated with active follicular growth. However, because hair-cycle stages were inferred from histological morphology rather than directly determined, these findings should be interpreted as supporting anagen-associated follicular activity rather than demonstrating a specific telogen-to-anagen transition.

At the molecular level, BEC treatment increased β-catenin expression, inhibitory phosphorylation of GSK-3β (Ser9), the p-AKT/AKT ratio, and PCNA expression in mouse dorsal skin. These findings indicate that the hair growth response to BEC was accompanied by changes in GSK-3β/β-catenin-, AKT-, and proliferation-related markers. Furthermore, inhibitor-based experiments in human follicle dermal papilla cells (HFDPCs) supported the functional involvement of AKT signaling in the cellular response to BEC. In contrast, functional inhibition or genetic interference of the GSK-3β/β-catenin pathway was not performed; therefore, its contribution should be interpreted as an associated molecular response rather than a confirmed causal mechanism.

Minoxidil was included as a positive control at 20 μg/mL. Because this concentration is substantially lower than that used in conventional clinical topical formulations, comparisons between BEC and MXD should be limited to the experimental conditions of the present study and should not be interpreted as evidence of equivalence or superiority to standard clinical minoxidil treatment. In addition, the absence of significant changes in body weight provides only limited information regarding tolerability and does not establish systemic safety. Comprehensive systemic toxicity assessments, including histopathological evaluation of major organs, pharmacokinetic analysis, and long-term toxicity studies, were not performed. Further studies incorporating these assessments are required to more comprehensively evaluate the systemic safety of BEC.

Several limitations should be acknowledged. Only a single BEC concentration (20 μg/mL) was evaluated in vivo, and an esculetin-free biorenovation filtrate control was not included in the animal study. Therefore, an in vivo dose–response relationship could not be determined, and potential contributions of the biorenovation process or residual medium-derived components cannot be completely excluded. The relatively small group size (*n* = 5) may also limit statistical power. In addition, outcome assessments were not performed under blind conditions, which may have introduced potential observer bias. The causal contribution of the GSK-3β/β-catenin pathway remains to be established. Further studies incorporating appropriate process controls, multiple BEC concentrations, larger cohorts, blinded outcome assessment, and functional validation of the GSK-3β/β-catenin pathway are needed to further define the biological activity and mechanism of BEC.

## 4. Materials and Methods

### 4.1. Preparation of BEC

The biorenovation process was performed using the *Bacillus siamensis* JD3-7 strain (KACC 92346P), which was isolated from traditional soy sauce from Jeju Island. The strain was cultured in Luria–Bertani broth (LB, BD Biosciences, San Jose, CA, USA) at 30 °C with shaking at 200 rpm for 16 h. After cultivation, the supernatant was removed by centrifugation (4416× *g*, 15 min, 4 °C), and the cell pellet was collected. The collected cells were washed and suspended in PG buffer (50 mM phosphate buffer, 2% glycerin), after which 100 mg of esculetin was added. The biotransformation reaction was carried out at 30 °C and 200 rpm for 72 h. After the reaction was complete, the supernatant was collected by centrifugation, and ethyl acetate (EA, Samchun Chemicals, Seoul, Republic of Korea) was added at twice the sample volume to separate the aqueous and organic layers. The aqueous layer was collected and subjected to vacuum concentration to remove residual EA, filtered through an SPL 0.22 µm bottle-top filter (SPL Life Sciences, Pocheon-si, Gyeonggi-do, Republic of Korea) to eliminate microorganisms and impurities, and freeze-dried to obtain *Bacillus*/Esculetin Ferment Filtrate (BEC) powder. The chemical profile of BEC was further characterized by HPLC and LC–MS, and its refrigerated storage stability at 4 °C was evaluated by HPLC for 8 weeks, as described in the Supplementary Methods (Supplementary Figures S1 and S2; Table S1).

### 4.2. Animals and Treatment

Five-week-old male C57BL/6N mice (*n* = 15) were purchased from JA BIO (Suwon, Republic of Korea) and acclimatized for 7 days. The animals were allocated to three groups (*n* = 5 per group): vehicle control, MXD-treated group (20 μg/mL), and BEC-treated group (20 μg/mL). Animals were housed according to treatment group and in-dividually identified by number (1–5) within each cage for treatment and sample tracking. A formal a priori power analysis was not performed. The sample size of five animals per group was selected considering the exploratory nature of the study, experimental feasibility, and ethical considerations regarding the reduction in animal use. The BEC concertation was selected based on the biological activity observed in prior in vitro evaluations and considerations related to topical administration. After anesthesia with isoflurane, the dorsal skin of each mouse was shaved over an area of approximately 2 × 4 cm. Beginning on the day after depilation, 40 mg of the assigned topical preparation was applied once daily for 24 days. Treatment administration and outcome assessments, including hair measurements and histological quantification, were performed with knowledge of the treatment allocation; therefore, blinded outcome assessment was not implemented in the present study. All experimental procedures were approved by the Institutional Animal Care and Use Committee (IACUC) of Kyung Hee University (approval number: KHSASP-25-522). The study was conducted in accordance with the approved institutional animal care protocol and reported in accordance with the ARRIVE guidelines.

### 4.3. Measurement of Hair Thickness

On day 24 after shaving, the mice were euthanized, and ten mouse hairs were collected from the anterior, middle, and posterior regions of the depilated dorsal area of each mouse using forceps. Hair thickness was measured at the widest portion of each hair shaft using a CKX41 inverted microscope (Olympus Corporation, Tokyo, Japan) equipped with an eXcope imaging system (DIXI Science, Daejeon, Republic of Korea) and eXcope imaging software (version 3.7.11827.20180507). The measured values were recorded and used for quantitative analysis.

### 4.4. Measurement of Hair Length

On day 24 after shaving, the mice were euthanized, and ten hairs were plucked from the anterior, middle, and posterior regions of the shaved dorsal area of each mouse. Images were acquired at 20× magnification using an MSP^TM^ 320 imaging system (Curiosis Inc., Seoul, Republic of Korea). Hair length was measured from the original, unedited images using MSP-320 Image Data Viewer software (Version 1.0.4), with the 1 mm scale bar used as a reference. For representative presentation in the figures, the measured hairs were isolated from the original images, rotated when necessary for consistent orientation, and placed on a uniform background using Adobe Photoshop CS6 (version 13.0). These adjustments were performed solely for figure presentation and did not affect the original measurements or the morphology of the hairs.

### 4.5. Measurement of Hair Weight

On day 24 after shaving, hair was clipped from a defined 0.5 cm × 0.5 cm area of the dorsal skin of each mouse. The hair was clipped off with an electric clipper, and the hair weight was measured using an analytical balance (PX224KR/E, OHAUS Corporation, Parsippany, NJ, USA).

### 4.6. Histological Analysis

Dorsal mouse skin samples measuring 2 cm × 3 cm were collected. Following hair clipping, the tissues were fixed in 4% paraformaldehyde for 24 h, embedded in paraffin, and cut into sections for further analysis. The sections were deparaffinized and stained with hematoxylin and eosin. The number and depth of hair follicles were assessed using the EVOS M7000 Imaging System (Invitrogen, Carlsbad, CA, USA). Hair follicle number, follicle depth, and dermal thickness were quantified using ImageJ software (version 1.53t; NIH, Bethesda, MD, USA).

### 4.7. HFDPC Culture and AKT Inhibitor Treatment

Human follicle dermal papilla cells (HFDPCs) were provided bySeung-Young Kim (Sunmoon University, Asan, Republic of Korea). HFDPCs were maintained in Follicle Dermal Papilla Cell Growth Medium (C-26501) (PromoCell Gmbh, Heidelberg, Germany) according to the manufacturer’s instructions. To investigate the functional involvement of AKT signaling in the cellular response to BEC, HFDPCs were pretreated with the AKT inhibitor MK-2206 (1 μM) for 1 h. Following inhibitor pretreatment, cells were treated with BEC (10 μg/mL) for 30 min. Immediately after treatment, cells were harvested for protein extraction and subsequent Western blot analysis.

### 4.8. Western Blotting

After euthanasia, a 0.5 cm × 0.5 cm skin sample was excised from each mouse and homogenized in 1× phosphate-buffered saline (PBS) containing NP-40 buffer using a handheld homogenizer (MT-13K-L, MIULAB, Hangzhou, China) to extract proteins. The homogenates were centrifuged at 13,000 rpm for 10 min at 4 °C, and the protein concentration was determined at 750 nm using the Bio-Rad RC-DC Protein Assay (Bio-Rad, Hercules, CA, USA). For the HFDPC experiments, cells were harvested immediately after treatment and processed for protein extraction using an NP-40-based lysis procedure. Subsequently, 60 μg of protein from each sample was separated by SDS-PAGE (8–10%) and transferred to nitrocellulose membranes. The membranes were blocked with 5% skim milk and incubated overnight at 4 °C with the following primary antibodies diluted in 1× PBS containing 0.1% Tween 20: β-catenin (1:1000, #9562), phospho-GSK-3β (Ser9) (D85E12) (1:1000, #5558), GSK-3β (27C10) (1:1000, #9315), phospho-AKT (Ser473) (D9E) (1:1000, #4060), AKT (pan) (C67E7) (1:1000, #4691) *PCNA* (*D3H8P*) (1:1000, #13110) (Cell Signaling Technology Inc., Danvers, MA, USA), and β-actin (C4) (1:1000, sc-47778, Santa Cruz Biotechnology Inc., Dallas, TX, USA). After washing, the membranes were incubated with secondary antibodies: goat anti-rabbit IgG-HRP and goat anti-mouse IgG-HRP (cat. no. A24531 and sc-516102, respectively). Protein bands were visualized using an Image Quant LAS 500 (GE Healthcare Life Sciences, Uppsala, Sweden)), and band intensities were quantified using ImageJ software (version 1.53t; NIH, Bethesda, MD, USA).

### 4.9. Statistical Analysis

Data are presented as the mean ± standard deviation (SD). For comparisons among three or more groups, statistical significance was evaluated using one-way analysis of variance (ANOVA), followed by Dunnett’s multiple comparisons test. For animal experiments, statistical significance compared with the vehicle control group is denoted as * *p* < 0.05; ** *p* < 0.01; *** *p* < 0.001; **** *p* < 0.0001. Significance compared with the MXD-treated group is denoted as ^#^ *p* < 0.05; ^##^ *p* < 0.01; ^###^ *p* < 0.001; ^####^ *p* < 0.0001.

## 5. Conclusions

In conclusion, topical BEC treatment promoted hair regrowth in a C57BL/6 mouse model, as demonstrated by increases in hair weight, length, and thickness, together with increases in hair follicle number and depth. These changes were accompanied by modulation of GSK-3β/β-catenin-, AKT-, and proliferation-related markers in mouse dorsal skin.

In addition, inhibitor-based experiments in HFDPCs supported the functional involvement of AKT signaling in the cellular response to BEC, whereas the causal contribution of the GSK-3β/β-catenin pathway remains to be established. Collectively, these findings support the hair growth-promoting activity of BEC and provide a basis for further investigation of its underlying mechanisms and potential applications in hair regeneration.

## Data Availability

The original contributions presented in this study are included in the article. Further inquiries can be directed to the corresponding authors.

## Supplementary Materials

The following supporting information can be downloaded at https://www.mdpi.com/article/10.3390/ph19091430/s1: **Figure S1**: Chemical characterization of the major biorenovation product (P1) in BEC. (A) HPLC chromatogram showing the major biorenovation product (P1) detected in BEC. (B) LC-MS spectrum of P1. **Figure S2**: HPLC chromatograms of BEC during storage at 4 °C for 8 weeks. No noticeable changes in the chromatographic profiles were observed throughout the storage period, and no additional degradation peaks were detected. **Figure S3**: Effects of the esculetin-free fermentation control (EFC) on the proliferation of human Follicle dermal papilla cells (HFDPCs). HFDPCs were treated with EFC (1, 5, 10, 25, 50, 100, and 200 μg/mL) for 48 h under serum-starved conditions. MXD (10 μM) was used as a positive control. Data are expressed as the mean ± standard deviation (SD) (*n* = 3). * *p* < 0.05 vs. untreated control. MXD, minoxidil; EFC, esculetin-free fermentation control. **Figure S4**: Representative high-resolution images of mouse hair and quantitative analysis of hair length. (A) Representative images of mouse hairs imaged at 20× magnification using a fluorescence microscope. Mouse hairs were randomly collected from each experimental group, mounted on glass slides, and hair length was quantified using the MSP^TM^ 320 imaging system. Hair length was quantified with the MSP-320 Image Data Viewer software with the 1-mm scale bar as a reference. (B) Quantitative analysis of hair length in each group. Hair length was analyzed using GraphPad Prism 8.0. Data are presented as the mean ± SD (*n* = 4 per group). Statistical analysis was performed using ordinary one-way ANOVA, followed by Dunnett’s multiple comparisons test. ** *p* < 0.01; **** *p* < 0.0001 vs. vehicle control group. ^##^ *p* < 0.01 vs. MXD-treated group. **Figure S5**: Effects of MK-2206 on the BEC-induced AKT signaling in human follicle dermal papilla cells (HFDPCs). HFDPCs were pretreated with the AKT inhibitor MK-2206 (1 μM) for 1 h, followed by treatment with BEC (10 μg/mL) for 30 min. Cells were harvested immediately after treatment and subjected to Western blot analysis. (A) Representative Western blot images of p-AKT, total AKT, and β-actin. (B) Quantitative analysis of the p-AKT/AKT ratio. β-actin was used as a loading control. Data are expressed as the mean ± standard deviation (SD) (*n* = 3). * *p* < 0.05; *** *p* < 0.001 vs vehicle control group. ^####^ *p* < 0.0001 vs. MK-2206 inhibitor treated group. **Table S1**: Area percentage of the major biorenovation product (P1) during storage at 4 °C for 8 weeks.

## Abbreviations

The following abbreviations are used in this manuscript:

AKT	Protein Kinase B (PKB)
BEC	Biorenovated Esculetin Complex
GSK-3β	Glycogen Synthase Kinase-3 beta
MXD	Minoxidil
p-	Phosphorylated
PCNA	Proliferating Cell Nuclear Antigen
Wnt	Wingless-related integration site
β-catenin	Beta-catenin
