# Supplementary material for: Hair Growth-Promoting Activity of a Biorenovated Esculetin Complex Through Modulation of Dermal Papilla Cell Function and Hair Follicle Cycling
Source: Pharmaceuticals (Basel). 2026 Sep 10;19(9):1430. doi: 10.3390/ph19091430 (PMC13610410; doi:10.3390/ph19091430)
Supplement: Supplementary file 1 [file pharmaceuticals-19-01430-s001.zip › pharmaceuticals-4336987-supplementary.pdf]

Supplementary Materials

Supplementary Methods

HPLC Analysis

HPLC analysis was performed using a Shimadzu HPLC system (Shimadzu Corporation, Kyoto, Japan) equipped with a UV detector and a reversed-phase C18 column. Chromatographic separation was achieved using a water/acetonitrile gradient containing 0.1% trifluoroacetic acid, and detection was carried out at 345 nm.

LC-MS Analysis

LC–MS analysis was conducted using an ACQUITY ultra-performance liquid chromatography system coupled with a SYNAPT G2-Si high-resolution quadrupole time-of-flight mass spectrometer (Waters Corporation, Milford, MA, USA) operating in positive electrospray ionization mode.

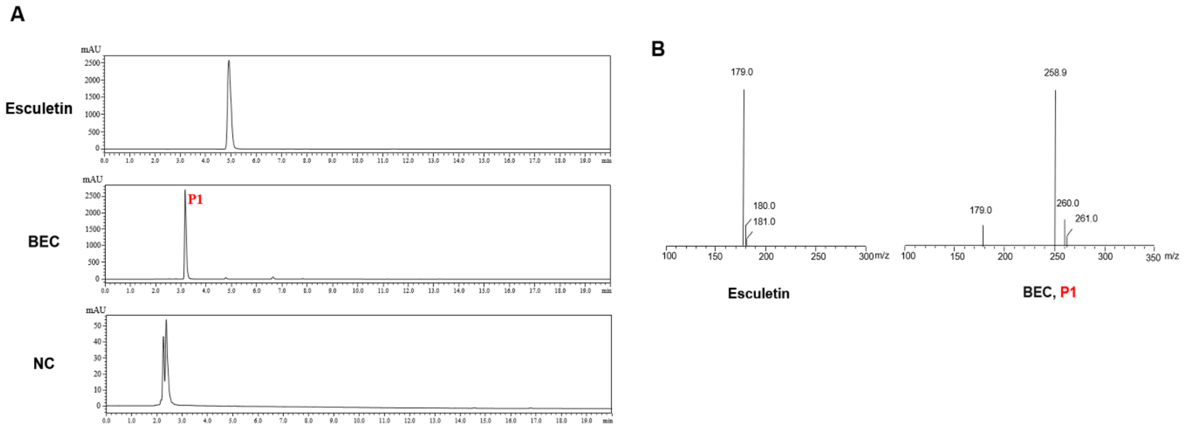

Figure S1. Chemical characterization of the major biorenovation product (P1) in BEC.

(A) HPLC chromatogram showing the major biorenovation product (P1) detected in BEC. (B) LC-MS spectrum of P1.

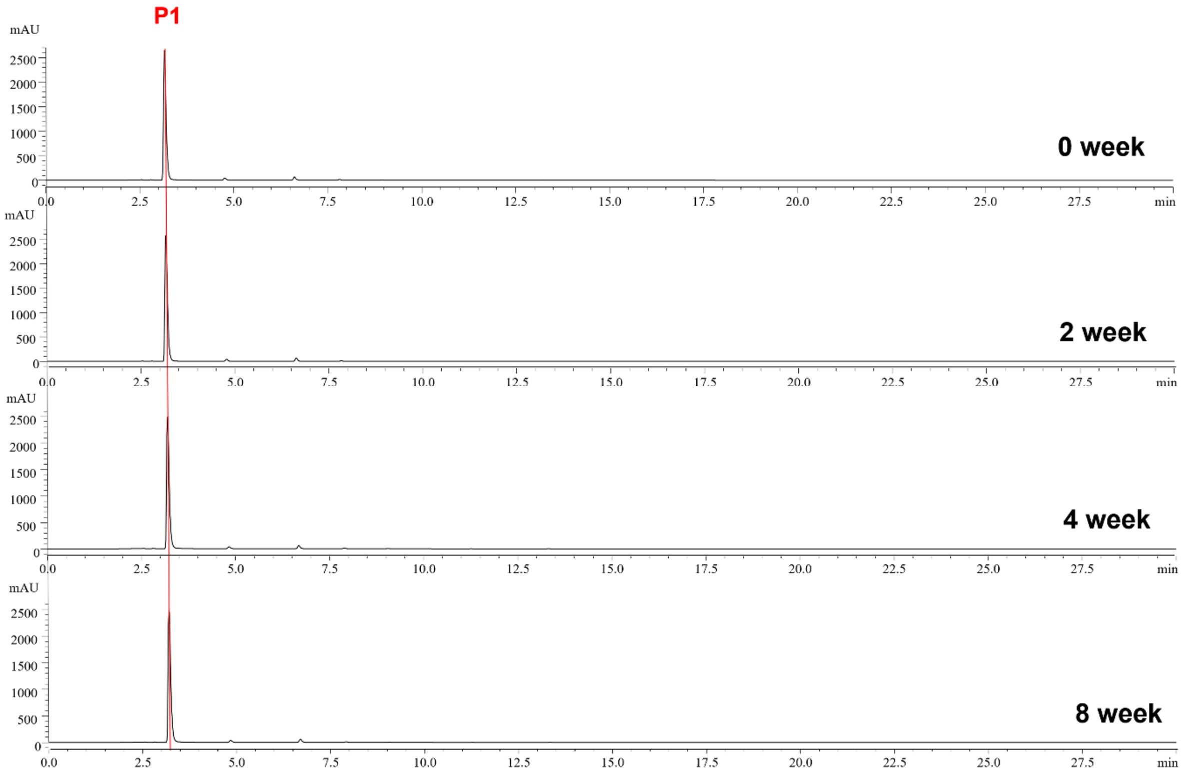

Figure S2. HPLC chromatograms of BEC during storage at 4°C for 8 weeks. No noticeable changes in the chromatographic profiles were observed throughout the storage period, and no additional degradation peaks were detected.

Time (week)	P1 peak area (%)
0	95.218
2	95.313
4	95.250
8	95.206

Table S1. Area percentage of the major biorenovation product (P1) during storage at 4°C for 8 weeks

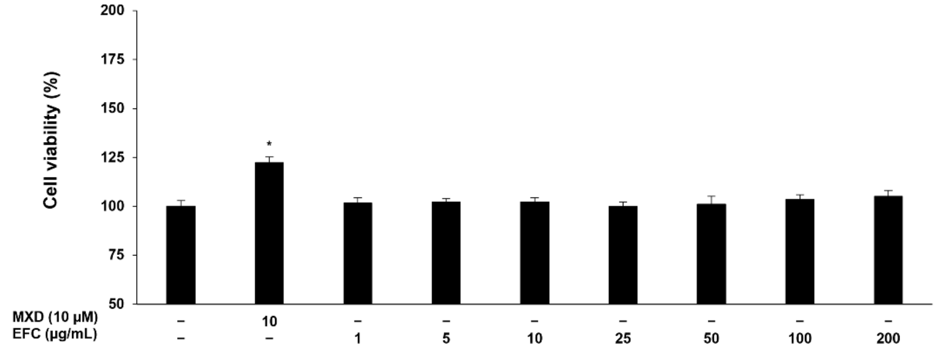

Figure S3. Effects of the esculetin-free fermentation control (EFC) on the proliferation of human Follicle dermal papilla cells (HFDPCs). HFDPCs were treated with EFC (1, 5, 10, 25, 50, 100, and 200 μg/mL) for 48 h under serum-starved conditions. MXD (10 μM) was used as a positive control. Data are expressed as the mean ± standard deviation (SD) (n = 3). *$p < 0.05$ vs. untreated control. MXD, minoxidil; EFC, esculetin-free fermentation control.

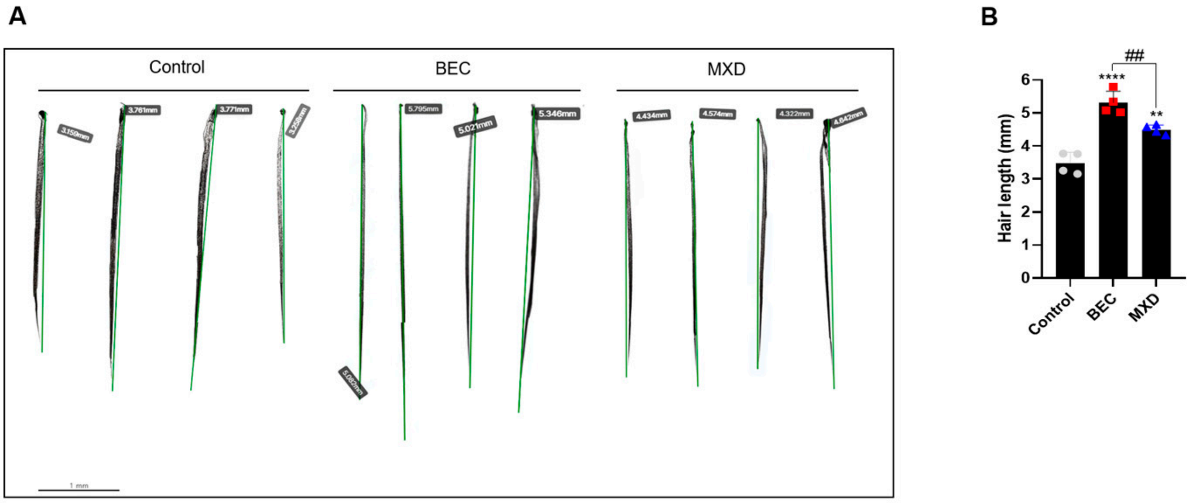

Figure S4. Representative high-resolution images of mouse hair and quantitative analysis of hair length. (A) Representative images of mouse hairs imaged at 20× magnification using a fluorescence microscope. Mouse hairs were randomly collected from each experimental group, mounted on glass slides, and hair length was quantified using the MSP^{TM} 320 imaging system. Hair length was quantified with the MSP-320 Image Data Viewer software with the 1-mm scale bar as a reference. (B) Quantitative analysis of hair length in each group. Hair length was analyzed using GraphPad Prism 8.0. Data are presented as the mean ± SD (n=4 per group). Statistical analysis was performed using ordinary one-way ANOVA, followed by Dunnett's multiple comparisons test. $^{**}p$ <0.01; ****p<0.0001 vs. vehicle control group. $^{\#\#}p$ <0.01 vs. MXD-treated group.

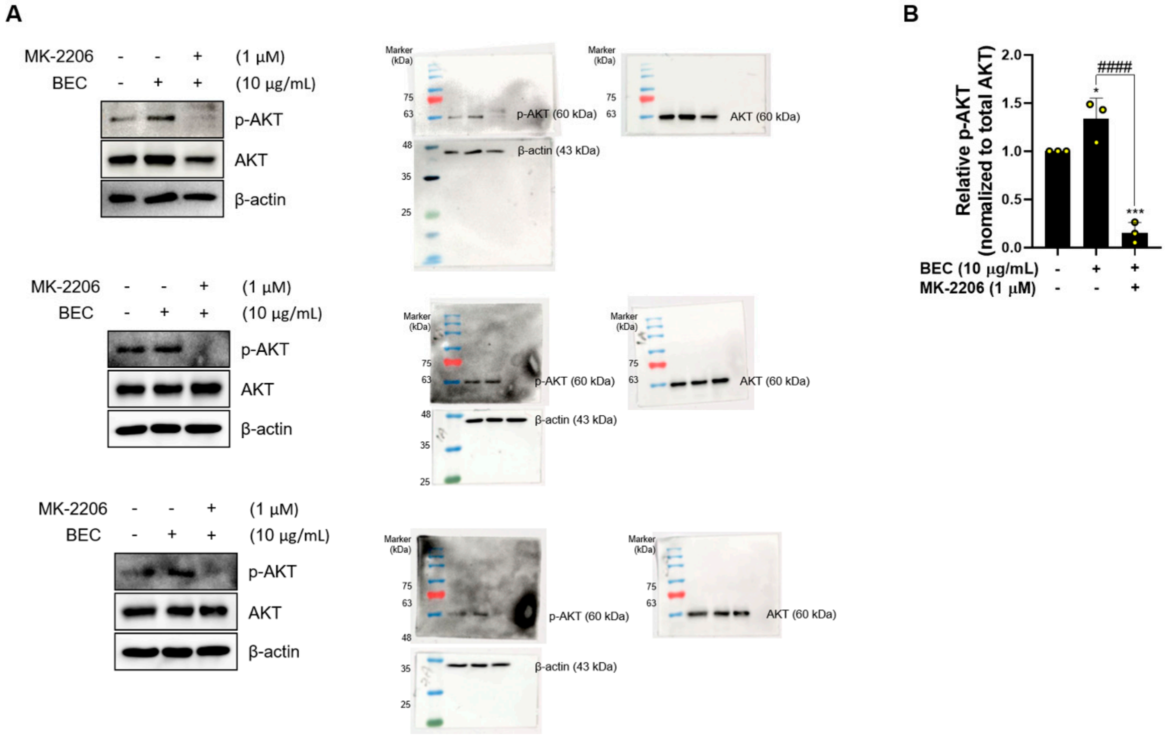

Figure S5. Effects of MK-2206 on the BEC-induced AKT signaling in human follicle dermal papilla cells (HFDPCs). HFDPCs were pretreated with the AKT inhibitor MK-2206 (1 μM) for 1 h, followed by treatment with BEC (10 μg/mL) for 30 min. Cells were harvested immediately after treatment and subjected to Western blot analysis. (A) Representative Western blot images of p-AKT, total AKT, and β-actin. (B) Quantitative analysis of the p-AKT/AKT ratio. β-actin was used as a loading control. Data are expressed as the mean ± standard deviation (SD) (n = 3). **p* < 0.05; ****p* <0.001 vs vehicle control group. ####*p* <0.0001 vs. MK-2206 inhibitor treated group.
